# Incidence and Predictors of Pulmonary Thromboembolism in Patients with Advanced High-Grade Serous Ovarian Cancer Undergoing Surgical Treatment: A Retrospective Cohort Study

**DOI:** 10.3390/jpm15070299

**Published:** 2025-07-09

**Authors:** Vito Andrea Capozzi, Michela Gaiano, Isabella Rotondella, Martina Leotta, Asya Gallinelli, Licia Roberto, Elisa Scarpelli, Carla Merisio, Roberto Berretta

**Affiliations:** Dipartimento di Ostetricia e Ginecologia, Università di Parma, 43125 Parma, Italy; vcapozzi@ao.pr.it (V.A.C.); isabella.rotondella@unipr.it (I.R.); martina.leotta@unipr.it (M.L.); asya.gallinelli@unipr.it (A.G.); licia.roberto@unipr.it (L.R.); elisascarpelli13@gmail.com (E.S.); carla.merisio@unipr.it (C.M.); rberretta@ao.pr.it (R.B.)

**Keywords:** ovarian carcinoma, venous thromboembolism, pulmonary thromboembolism chemotherapy, cytoreductive surgery, Khorana score

## Abstract

**Background/Objectives**: Patients with advanced ovarian cancer face a high risk of venous thromboembolism (VTE). This study evaluates the incidence and risk factors for pulmonary thromboembolism (PE) in patients with advanced high-grade serous ovarian carcinoma (HGSOC) undergoing primary treatment, with a focus on personalized risk stratification. **Methods**: A retrospective analysis was conducted on women with FIGO stage IIIA-IVB HGSOC treated at the University Hospital of Parma between January 2012 and May 2023. All patients underwent CT-based staging prior to primary treatment. When resectability was uncertain, diagnostic laparoscopy and the Fagotti score were performed. Based on cytoreductive potential, patients received either primary debulking surgery (PDS) followed by adjuvant chemotherapy (AC) or neoadjuvant chemotherapy (NACT) followed by interval debulking surgery (IDS) and AC. The Khorana score, a thromboembolic risk model, was calculated prior to chemotherapy. Logistic regression was used to assess the association between baseline characteristics and PE. **Results**: Among 167 HGSOC patients analyzed, 13 (7.8%) experienced PE. Among the 115 patients undergoing diagnostic laparoscopy, each 2-point increase in the Fagotti score above 8 raised PE risk by 76% (OR 1.76, *p* = 0.006, 95% CI: 1.17–2.63). Patients undergoing NACT-IDS had a significantly higher risk of PE (OR 4.04, 95% CI: 1.19–13.74, *p* = 0.02) than patients who underwent PDS. A Khorana score of 3 was an independent predictor of PE (OR 37.66, 95% CI: 2.43–582.36, *p* = 0.009). **Conclusions**: Based on our results, NACT followed by IDS or a Fagotti score greater than 8 were associated with increased PE risk in HGSOC patients. Khorana score was the strongest predictor of PE in HGSOC patients.

## 1. Introduction

Venous thromboembolism (VTE), including pulmonary embolism (PE) and deep vein thrombosis (DVT), is the second leading cause of death among ovarian cancer patients, after cancer progression itself, and is a significant predictor of poor outcomes and decreased quality of life [1,2,3]. Among gynecologic cancers, ovarian cancer has a higher risk of thromboembolic events compared to most other solid tumors of non-gynecologic origin [4,5], with cumulative incidence of VTE ranging from 3.2% to 15% at 30 days and from 5.2% to 23% at 2 years after diagnosis [6,7,8]. Several mechanisms have been proposed to explain the high VTE risk in ovarian cancer patients. First, eighty percent of women with HGSOC are diagnosed at an advanced stage [9]. These patients often present large abdominal masses and ascites, which can compress the pelvic veins and increase blood stasis [10,11]. Secondly, the aggressive surgical and chemotherapy treatments used for ovarian cancer likely contribute to high rates of thrombosis [12,13,14]. This situation occurs in the context of a neoplastic hypercoagulable state caused by vessel wall irritation, chronic inflammation, and thrombocytosis [15,16,17].

Various risk assessment models (RAMs) have been developed to evaluate the risk of venous thromboembolism (VTE). In the perioperative setting, the Caprini Risk Score is a standardized and widely used tool in clinical practice [18]. It assesses VTE risk by considering individual factors such as age, body weight, personal history of VTE, comorbidities, and surgery-related variables, including the type and duration of the procedure.

**Figure 1:**
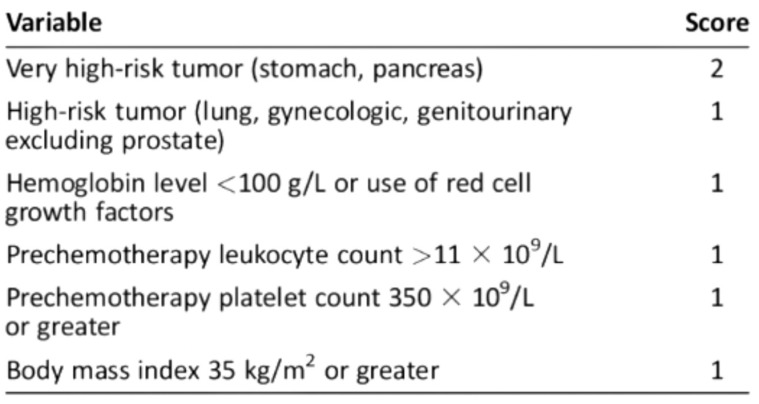
Khorana score: a predictive model for chemotherapy-associated thrombosis. Scoring and Risk Stratification: Low risk: 0 points, associated with a 0.3–1.5% risk of VTE; Intermediate risk: 1–2 points, associated with a 2.0–4.8% risk of VTE; High risk: ≥3 points, associated with a 6.7–12.9% risk of VTE.

For ambulatory cancer patients, the Khorana Score, assessed immediately prior to the initiation of chemotherapy (Figure 1), is recommended by the American Society of Clinical Oncology guidelines to guide decisions on thromboprophylaxis [19,20].

Despite ongoing discussions, clinicians are still working to develop a scoring system to identify outpatients who would benefit most from low-molecular-weight heparin (LMWH) prophylaxis. International guidelines currently lack a unified consensus on the criteria for administering thromboprophylaxis to cancer patients undergoing systemic therapy. The National Comprehensive Cancer Network (NCCN) [21] and the American Society of Clinical Oncology (ASCO) [19] strongly recommend thromboprophylaxis for high-risk cancer outpatients undergoing chemotherapy, highlighting the critical need for venous thromboembolism (VTE) prevention in this vulnerable population. The Associazione Italiana di Oncologia Medica (AIOM) [22] supports this recommendation but calls for more research on VTE risks across different cancer types and treatment settings. In contrast, the American Society of Hematology (ASH) [23] expresses concerns about the potential for increased bleeding. Therefore, ASH does not recommend routine preventive treatment for low- or intermediate-risk patients due to insufficient evidence. The European Society for Medical Oncology (ESMO) [24] suggests considering preventive treatment for patients with more than an 8% to 10% VTE risk over six months, promoting a balanced and personalized approach.

In summary, international guidelines currently lack consensus on which cancer patients should receive thromboprophylaxis during systemic therapy. The lack of standardized and personalized protocols for VTE prevention in advanced ovarian cancer during chemotherapy highlights a critical gap in clinical practice.

The primary aim of this study was to determine the incidence of PE in our cohort of patients with HGSOC. Additionally, we aimed to identify risk factors to help define a higher-risk subgroup that may benefit from preventive thromboprophylaxis.

## 2. Materials and Methods

### 2.1. Study Design

The Strengthening the Reporting of Observational Studies in Epidemiology (STROBE) guidelines were used for this retrospective cohort study [25]. A monocentric observational retrospective cohort study of women diagnosed with advanced ovarian, fallopian tube, and peritoneal serous high-grade carcinoma at the University Hospital of Parma (Italy) between January 2012 and May 2023 was performed. Demographic and clinico-pathologic factors were analyzed as potential predictors of VTE. All data were retrospectively extracted from prospectively maintained electronic medical records on REDCap and reviewed by three independent authors (M.G., I.R., M.L.). The study was approved by the Ethics Committee of the University of Parma (code 6603/16082023/OSS/AOUPR). Informed consent was obtained from all living participants who were able to provide it; for deceased patients, data collection complied with GDPR Regulation No. 679/2016 and Legislative Decree 196/2003.

### 2.2. Patient Selection and Treatment

Adult patients with advanced HGSOC (histologically confirmed stage III or IV according to FIGO) treated at the University Hospital of Parma were included in the analysis. Patients with a personal history of a VTE event before their oncological diagnosis were excluded.

According to Enhanced Recovery After Surgery (ERAS^®^) society guidelines, patients at an increased risk of venous thromboembolism (VTE) received dual prophylaxis, which includes both mechanical compression and chemoprophylaxis, starting in the preoperative phase. In order to reduce the risk of bleeding in the immediate postoperative period, LMWH administration was resumed 12 h after the completion of surgery. The postoperative thromboprophylaxis protocol with LMWH was standardized to a 28-day regimen based on the ERAS^®^ guidelines assessment and was uniformly applied to all patients in the study cohort [26].

Furthermore, early ambulation following surgery was strongly encouraged as an integral component of our institutional enhanced recovery protocols [27]. No patients received thromboprophylaxis during NACT treatment or CT treatment.

### 2.3. Covariates

Demographic, preoperative, intraoperative and postoperative data were collected [see Table 1]. Surgical complexity was assessed using Surgical Complexity Score System (SCSS) described by Aletti et al., and postoperative complications were classified according to the Clavien–Dindo grading system [26,28].

PE occurrence was analyzed during three phases: between PDS and adjuvant chemotherapy (AC), between NACT and IDS, and between IDS and subsequent AC, up to the end of chemotherapy treatments. The perioperative period was defined as the time from the last NACT cycle to surgery and/or from surgery to the first AC cycle.

All patients with a suspicion of advanced HGSOC underwent staging with contrast-enhanced CT scan. Patients with uncertain resectability on imaging, based on tumor load and disease spread, underwent minimally invasive procedures to assess cytoreducibility. Patients were deemed eligible for PDS with a Fagotti score ≤ 8 and in the absence of inoperability criteria [29]. NACT candidates, considered not immediately suitable for cytoreduction, had a Fagotti score greater than 8 or met inoperability criteria such as extensive peritoneal carcinomatosis involving the mesenteric root, unresectable disease encasing major vessels, or poor performance status precluding major surgery as determined by multidisciplinary evaluation [30].

**Figure 2:**
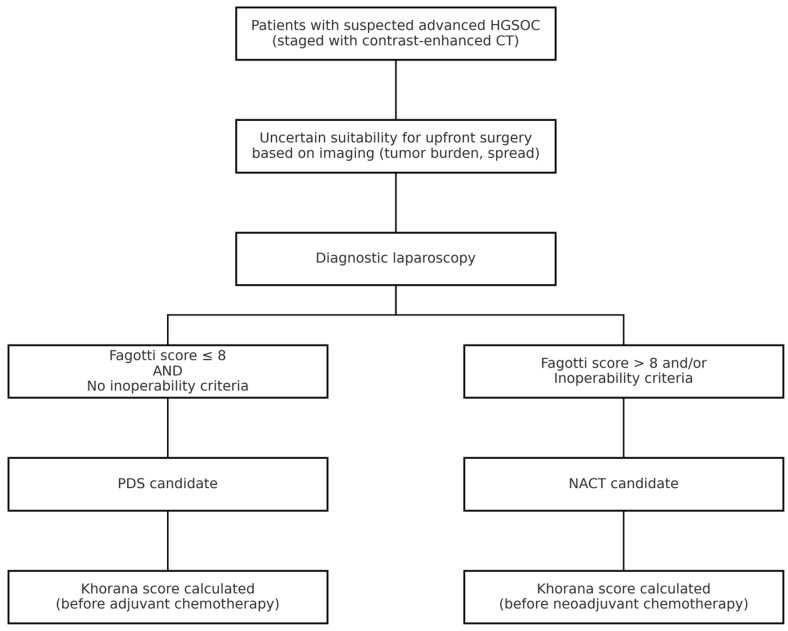
Diagnostic and therapeutic workflow for patients with suspected advanced HGSOC. CT = computed tomography; NACT = neoadjuvant chemotherapy.

The Khorana score was analyzed based on platelet, white blood cell, and hemoglobin values before the first cycle of chemotherapy (Figure 2) [20].

PE was defined and classified according to the 2019 ESC Guidelines, which categorize events as mild, submassive, or massive based on hemodynamic stability, right ventricular dysfunction, and biomarker levels [31]. Both symptomatic and asymptomatic PE events were considered. All diagnoses were confirmed via contrast-enhanced CT. No routine screening was performed, and asymptomatic cases were identified incidentally during CT scans conducted for disease monitoring in the context of HGSOC follow-up. The follow-up period extended through completion of first-line chemotherapy.

### 2.4. Statistical Analysis

Descriptive statistics were performed using frequencies and percentages for categorical data and mean with standard deviation (SD) for continuous variables. The two groups of patients who did or did not experience PE were compared by clinicopathological characteristics using the T-test, Mann–Whitney U test, and Chi-square test, as appropriate. Univariate and multivariate binomial logistic regression models were fit to evaluate the association between baseline characteristics and PE. The regression coefficients expressed as odds ratios enable the interpretation of the impact of the independent variables on the event of interest. In the multivariate analysis, we assessed the simultaneous effects of different confounding values. Variables were selected based on their statistical significance in univariate analyses. All calculated *p* values were two-sided and *p* values < 0.05 were considered statistically significant. The statistical analysis was performed using Jamovi (The Jamovi Project, 2021-Version 2.5.3) [32].

## 3. Results

**Figure 3:**
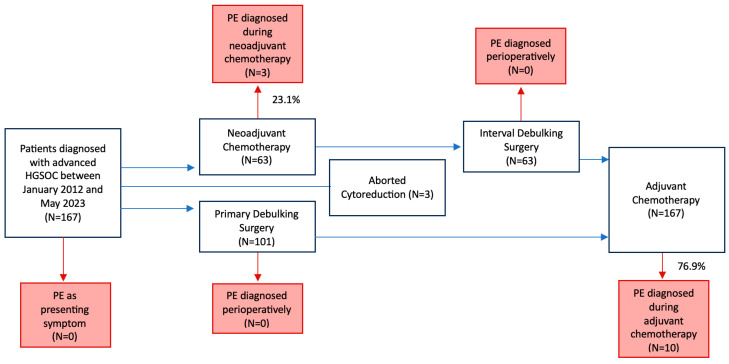
Study flow diagram. PE, Pulmonary thromboembolism.

Over the study period, 167 HGSOC patients met the selection criteria. Figure 3 provides a schematic representation of the study flow.

In total, 13 (7.8%) PEs were observed. Of these, 3 (23.1%) occurred during NACT, 10 (76.9%) during AC, and none in the perioperative period. Among the 13 PEs recorded, 6 (46.2%) were of mild severity, 6 (46.2%) were submassive, and 1 (7.6%) was massive. Additionally, 8 (61.5%) of these events were associated with deep vein thrombosis (DVT). Five (38.5%) patients showed clinically relevant symptoms, while eight (61.5%) were without symptoms. However, 46.2% of cases required hospitalization, while none resulted in patient death (Table 2).

Patient characteristics stratified by the occurrence of PE are reported in Table 3. Mean age (62.7 ± 11.5 years) and mean BMI (24.6 ± 5.0 kg/m^2^) did not significantly differ between the two groups of patients who did or did not experience a thromboembolic event (*p* = 0.45 and *p* = 0.89, respectively).

Ascites at diagnosis were present in 42.5% of patients and were associated with a higher incidence of PE compared to patients without ascites (12.7% vs. 4.2%, *p* = 0.042). The presence of postoperative complications, classified according to Clavien–Dindo, showed a significantly different distribution in patients with PE (*p* = 0.024).

PDS followed by AC was associated with a lower risk of PE compared to NACT followed by IDS (4% vs. 14.3%, *p* = 0.049).

Additionally, during chemotherapy, the Khorana score was significantly associated with PE: 1.3% of PE in Khorana score 1, 10.8% in Khorana score 2 and 25% in Khorana score 3 (*p* = 0.003).

**Figure 4:**
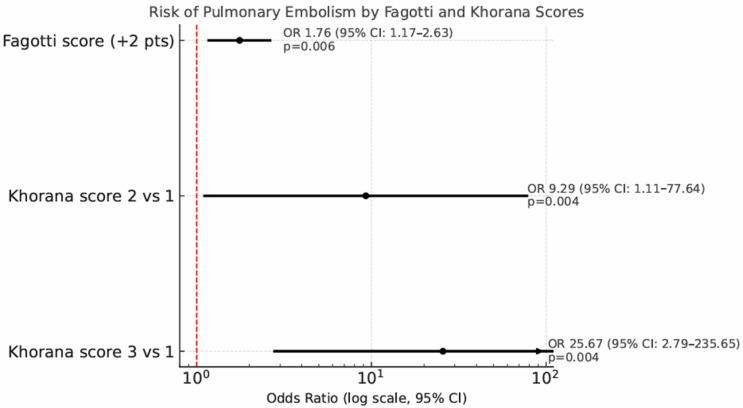
Forest plot showing the risk of pulmonary embolism (PE) by Fagotti and Khorana scores. Odds ratios (ORs) with 95% confidence intervals are reported on a logarithmic scale.

Univariate and multivariate analyses for predictors of PE are showed in Table 4. Patients undergoing IDS show a significantly higher risk of PE compared to those undergoing PDS, with an odds ratio of 4.04 (95% CI: 1.19–13.74, *p* = 0.02). Out of the 115 patients who underwent diagnostic laparoscopy before debulking surgery, each 2-point increase in the Fagotti score was associated with a 76% increased risk of developing PE (odds ratio 1.76, *p* = 0.006, 95% CI: 1.17–2.63). The risk of PE correlates significantly with the Khorana score: compared to Khorana score 1, a Khorana score of 2 raises the PE risk with an odds ratio of 9.29 (95% CI: 1.11–77.64, *p* = 0.004), while a Khorana score of 3 increases the risk with an odds ratio of 25.67 (95% CI: 2.79–235.65, *p* = 0.004) (Figure 4).

In the multivariate analysis, only Khorana score 3 (OR 37.66, 95% CI 2.43–582.36, *p* = 0.009) was associated with an increased risk of PE.

## 4. Discussion

### 4.1. Summary of Main Results

In our retrospective cohort, the incidence of PE among patients with advanced HGSOC was 7.8%, with most events occurring during chemotherapy, particularly during adjuvant treatment, rather than in the perioperative period.

Patients who received NACT followed by IDS faced a significantly elevated risk of PE compared to those undergoing PDS followed by adjuvant chemotherapy (OR 4.04, 95% CI: 1.19–13.74, *p* = 0.02). Among patients assessed with diagnostic laparoscopy, each 2-point increase in the Fagotti score above 8 was associated with a 76% rise in PE risk (OR 1.76, 95% CI: 1.17–2.63, *p* = 0.006).

Importantly, a Khorana score of 3 emerged as the only independent predictor of PE in the multivariate analysis (OR 37.66, 95% CI: 2.43–582.36, *p* = 0.009), underscoring its potential value in identifying high-risk patients.

### 4.2. Results in the Context of Published Literature

Specific data on the incidence, characteristics, and risk factors for PE in ovarian cancer are limited, making it challenging to compare previous studies with our cohort, as most of the existing literature focuses more broadly on VTE [33]. In our cohort, PE occurred in 7.8% of patients, most frequently during chemotherapy, particularly in the adjuvant phase, and was more common among those treated with NACT followed by IDS compared to PDS. Previous studies have described an increased risk of PE in women with advanced-stage ovarian cancer undergoing NACT followed by IDS, particularly during chemotherapy, consistent with our observations. Salinaro et al. (2020) identified 16 VTE events (7.7%) during NACT in patients with advanced epithelial ovarian cancer, of which 8 (3.8%) were pulmonary embolisms [34]. Similarly, Basaran et al. (2021) documented a 25.9% incidence of VTE during NACT, including 10 cases of isolated PE (4.3%) and 6 cases of combined DVT and PE (2.6%) [35]. These data only partially reflect our observation, as in our cohort PE occurred predominantly during the adjuvant phase in IDS patients. This difference may be attributed to heterogeneity in patients’ baseline characteristics and treatment selection criteria for NACT.

Although surgery is a recognized prothrombotic factor, most PE events in our cohort occurred during chemotherapy—particularly in the adjuvant setting—rather than in the perioperative phase. This observation aligns with evidence linking platinum-based chemotherapy to increased thrombotic risk, as confirmed in multiple oncology settings [1,36]. As PE remains one of the leading non-cancer-related causes of death in ovarian cancer, understanding its timing is critical for guiding thromboprophylaxis strategies.

Interestingly, in our study, surgical variables such as surgical complexity score (SCSS), hospital stay duration, perioperative blood transfusion, length of surgery, and postoperative complications were not significantly associated with PE risk. This diverges from prior reports such as Mokri et al.’s (2022), which found a 6.5% incidence of postoperative VTE following PDS, with isolated PE accounting for over 70% of these cases [37]. However, these studies were conducted before the widespread use of dual thromboprophylaxis and LMWH prophylaxis, as currently recommended.

A novel finding of our study is the association between higher Fagotti scores and PE risk in patients undergoing diagnostic laparoscopy. While the Fagotti score is well-established as a predictor of resectability in advanced ovarian cancer [29] to our knowledge, no prior study has linked it to thromboembolic complications. This association may reflect the impact of greater tumor burden and disease distribution on thrombosis risk. Although this variable did not retain statistical significance in the multivariate analysis, its association in the univariate analysis warrants further investigation.

Lastly, our findings support the prognostic utility of the Khorana score in this population. While its predictive performance in gynecologic malignancies has been questioned in prior studies [35], we found that a Khorana score of 3 was independently associated with a significantly increased risk of PE (OR 37.66). Consistent with major international guidelines [19,21,24], our analysis reinforces the validity of the high-risk Khorana score [20] as an independent predictor of PE events.

### 4.3. Strengths and Weaknesses

Our study’s strength lies in its in-depth analysis of PE incidence and risk factors in patients receiving extended thromboprophylaxis per current guidelines. Another key strength of our study is the comprehensive follow-up of all patients from diagnosis through completing their primary treatment course. This approach provided an inclusive and detailed analysis of all phases associated with a heightened risk of thrombosis, ensuring a thorough understanding of PE risk during the treatment process.

As a single-institutional and retrospective study, we have limitations and potential biases. First, the number of samples was limited and the relatively small number of thromboembolic events limits the ability to draw definitive conclusions about potential risk factors. Second, some PE events might have gone undetected, as our analysis was limited to documented and recorded cases in our radiology reporting system. Moreover, our findings are further limited by the absence of data regarding smoking status and concurrent therapies administered during NACT and CHT, including antiplatelet agents, erythropoietic growth factors, antiangiogenic maintenance treatments, PARP inhibitors, or deviations from standard chemotherapy protocols. Additionally, we did not assess the prognostic impact of PE events on disease progression, focusing instead solely on a descriptive analysis of the clinical characteristics associated with the onset of PE.

### 4.4. Implications for Practice and Future Research

There is a critical and urgent need to investigate the clinically relevant PE risk factors for ovarian cancer patients to compile a comprehensive understanding of those most vulnerable to thrombotic events. The widespread adoption of the Fagotti score as an index of non-cytoreducibility provides a valuable tool for further scientific investigations, particularly in optimizing thromboembolic risk management in outpatients undergoing chemotherapy, especially in the neoadjuvant setting.

Future research should focus on integrating the Fagotti score and other predictive markers into personalized anticoagulation decision-making tools. These tools could facilitate early identification of high-risk patients, enabling tailored prophylactic strategies and improving patient outcomes. Additionally, investigating the interplay between tumor biology, systemic inflammation, and hypercoagulability in ovarian cancer could refine risk stratification models and lead to the development of precision medicine approaches.

By advancing personalized risk assessment and thromboprophylaxis strategies, these findings have the potential to reduce thrombosis-related mortality and improve the overall management of ovarian cancer patients, aligning with the principles of individualized patient care.

## 5. Conclusions

Patients with HGSOC undergoing NACT followed by IDS exhibited a significantly higher risk of PE compared to those undergoing PDS. A Fagotti score of 8 or higher emerged as a key risk factor for PE, reinforcing its role in both surgical decision-making and thromboembolic risk stratification. Moreover, a Khorana score of 3 was identified as an independent predictor of PE, highlighting its potential utility in guiding personalized thromboprophylaxis strategies. These findings underscore the need for tailored risk assessment models to optimize anticoagulation management and improve outcomes in this high-risk patient population.

## Figures and Tables

**Table 1:** Patient demographics and clinicopathological data.

Variable	Total n = 167 (%)
Age, years (mean, sd)	62.7 (11.5)
BMI, kg/m^2^ (mean, sd)	24.6 (5.0)
ASA 1 2 3	9 (5.4) 97 (58.1) 61 (36.5)
BRCA WT Mut Unknown	105 (62.9) 44 (26.3) 18 (18.8)
CA 125 (mean, sd)	1471 (2743)
Ascites No Si	96 (57.5) 71 (42.5)
FIGO Stage IIIA1 IIIA2 IIIB IIIC IVA IVB	5 (3) 1 (0.6) 16 (9.6) 110 (65.9) 11 (6.6) 24 (14.4)
Treatment Strategy PDS + AC NACT + IDS Aborted cytoreduction	101 (60.5) 63 (37.3) 3 (1.8)
Fagotti Score (n = 115) 2 4 6 8 10 12	4 (3.5) 13 (11.3) 25 (21.7) 31 (27.0) 34 (29.6) 8 (7.0)
Surgical Approach LPS LPT	5 (3) 162 (97)
SCSS 1 2 3	18 (10.8) 72 (43.1) 77 (46.1)
RT No residual disease Optimal cytoreduction Suboptimal cytoreduction	128 (76.6) 26 (15.6) 13 (7.8)
ICU, days (mean, sd)	1.6 (2.3)
Hospital stays, days (mean, sd)	9.8 (2.0)
Intraoperative blood loss, mL (mean, sd)	562 (507.0)
Operative time, min (mean, sd)	224 (79.4)
Blood transfusion No Yes	113 (67.7) 54 (32.3)
Clavien-Dindo 0 1 2 3a 3b 4a 4b	74 (44.3) 24 (14.4) 47 (28.1) 11 (6.6) 5 (3.0) 5 (3.0) 1 (0.6)
Khorana Score 1 2 3 4	78 (46.7) 65 (38.9) 20 (12) 4 (2.4)
*Abbreviations: BMI, Body Mass Index; sd, standard deviation; FIGO, International Federation of Gynecology and Obstetrics; WT, Wild Tipe; Mut, Mutated; PDS, Primary Debulking Surgery; IDS, Interval Debulking Surgery; AC, Adjuvant Chemotherapy; NACT, Neoadjuvant Chemotherapy; LPS, Laparoscopy; LPT, Laparotomy; SCSS, Surgical Complexity Score System; RT, Residual Tumor—Optimal cytoreduction < 1 cm—Suboptimal cytoreduction > 1 cm; ICU, Intensive Care Unit*	

**Table 2:** Characteristics of PE.

	Timing PE	
	NACT N = 3 (23.1)	Adjuvant CHT N = 10 (76.9)
**PE severity** **Mild** **Submassive** **Massive**	2 (66.7) 1 (33.3) 0	4 (40) 5 (50) 1 (10)
**PE symptoms** **No** **Yes**	3 (100) 0 (0)	5 (50) 5 (50)
**PE hospitalization** **No** **Yes**	1 (33.3) 2 (66.7)	6 (60) 4 (40)
**PE death** **No** **Yes**	3 (100) 0	10 (100) 0
*Abbreviations: PE, Pulmonary thromboembolism; NACHT, Neoadjuvant chemotherapy; CHT, chemotherapy*		

**Table 3:** Characteristics of the study cohort stratified according to the occurrence of pulmonary thromboembolism.

Variable	Patients			
		**Pulmonary Thromboembolism**		
	Total n = 167	No n = 154	Yes n = 13	*p* value
Age, years (mean, sd)	62.7 (11.5)	62.6 (11.7)	63.1 (9.2)	0.89
BMI, kg/m^2^ (mean, sd)	24.6 (5.0)	24.5 (4.9)	25.6 (6.7)	0.45
ASA 1 2 3	9 (5.4) 97 (58.1) 61 (36.5)	9 (100) 89 (91.8) 56 (91.8)	0 (0) 8 (8.2) 5 (8.2)	0.67
BRCA WT Mut Unknown	105 (62.9) 44 (26.3) 18 (18.8)	98 (93.3) 39 (88.6) 17 (64.4)	7 (5.6) 5 (11.4) 1 (5.6)	0.26
CA 125 (mean, sd)	1471 (2743)	1417 (2763)	2174 (2593)	0.09
Ascites No Yes	96 (57.5) 71 (42.5)	92 (95.8) 62 (87.3)	4 (4.2) 9 (12.7)	0.042
FIGO Stage IIIA1 IIIA2 IIIB IIIC IVA IVB	5 (3) 1 (0.6) 16 (9.6) 110 (65.9) 11 (6.6) 24 (14.4)	5 (100) 1 (100) 16 (100) 100 (90.9) 9 (81.8) 23 (95.8)	0 (0) 0 (0) 0 (0) 10 (9.1) 2 (18.2) 1 (4.2)	0.52
Treatment Strategy PDS + AC NACT + IDS Aborted cytoreduction	101 (60.5) 63 (37.3) 3 (1.8)	97 (96.0) 54 (85.7) 3 (100)	4 (4.0) 9 (14.3) 0 (0)	0.049
Fagotti Score (n = 115) (median, IR)	8 (2.5)	8 (2.5)	10 (1.3)	0.067
Surgical Approach LPS LPT	5 (3) 162 (97)	4 (80) 150 (92.6)	1 (20) 12 (7.4)	0.42
SCSS 1 2 3	18 (10.8) 72 (43.1) 77 (46.1)	16 (88.9) 67 (93.1) 71 (92.2)	2 (11.1) 5 (6.9) 6 (7.8)	0.84
RT No residual disease Optimal cytoreduction Suboptimal cytoreduction	128 (76.6) 26 (15.6) 13 (7.8)	120 (93.8) 23 (88.5) 11 (84.6)	8 (6.3) 3 (11.5) 2 (15.4)	0.37
ICU, days (mean, sd)	1.6 (2.3)	1.6 (2.2)	1.8 (2.9)	0.71
Hospital stay, days (mean, sd)	9.8 (2.0)	9.8 (1.9)	9.2 (2.5)	0.32
Intraoperative blood loss, ml (mean, sd)	562 (507.0)	568 (516.2)	488 (396.9)	0.59
Operative time, min (mean, sd)	224 (79.4)	225 (77.6)	222 (102)	0.91
Blood transfusion No Yes	113 (67.7) 54 (32.3)	106 (93.8) 48 (88.9)	7 (6.2) 6 (11.1)	0.27
Clavien-Dindo 0 1 2 3a 3b 4a 4b	74 (44.3) 24 (14.4) 47 (28.1) 11 (6.6) 5 (3.0) 5 (3.0) 1 (0.6)	69 (93.2) 22 (91.7) 44 (93.6) 9 (81.8) 5 (100) 5 (100) 0 (0)	5 (6.8) 2 (8.3) 3 (6.4) 2 (18.2) 0 (0) 0 (0) 1 (100)	0.024
Khorana Score 1 2 3 4	78 (46.7) 65 (38.9) 20 (12) 4 (2.4)	77 (98.7) 58 (89.2) 15 (75) 4 (100)	1 (1.3) 7 (10.8) 5 (25.0) 0 (0)	0.003
*Abbreviations: BMI, Body Mass Index; sd, standard deviation; FIGO, International Federation of Gynecology and Obstetrics; WT, Wild Tipe; Mut, Mutated; PDS, Primary Debulking Surgery; IDS, Interval Debulking Surgery; AC, Adjuvant Chemotherapy; NACT Neoadjuvant Chemotherapy; LPS, Laparoscopy; LPT, Laparotomy; SCSS, Surgical Complexity Score System; RT, Residual Tumor—Optimal cytoreduction < 1 cm—Suboptimal cytoreduction > 1 cm; ICU, Intensive Care Unit*				

**Table 4:** Predictors of Pulmonary Thromboembolism: univariate and multivariate analysis.

Variable	Predictors of PE			
	**Univariate Analysis**		**Multivariate Analysis**	
	OR (95% CI)	*p* value	OR (95% CI)	*p* value
Age, years	0.99 (0.95–1.05)	0.91		
BMI, kg/m^2^	0.96 (0.95–1.05)	0.4		
ASA 1 2 3	REF - -	0.99 0.99		
BRCA WT Mut Unknown	REF 1.79 (0.53–5.99) 0.82 (0.09–7.12)	0.34 0.86		
CA 125	1.00 (1.00–1.00)	0.3		
Ascites No Yes	REF 3.34 (0.98–11.31)	0.053		
Treatment Strategy PDS + AC NACT + IDS Aborted cytoreduction	REF 4.04 (1.19–13.74) -	0.02 0.99	5.51 (0.38–79.3) -	0.21 -
Fagotti Score (n = 115)	1.76 (1.17–2.63)	0.006	1.58 (0.88–2.82)	0.125
Surgical Approach LPS LPT	3.12 (0.32–30.2) REF	0.32		
SCSS 1 2 3	REF 0.59 (0.10–3.36) 0.67 (0.12–3.66)	0.59 0.67		
RT No residual disease Optimal cytoreduction Suboptimal cytoreduction	REF 1.96 (0.48–7.93) 2.72 (0.51 –14.46)	0.35 0.24		
ICU, days	1.04 (0.83–1.32)	0.71		
Hospital stay, days	0.89 (0.70–1.12)	0.32		
Intraoperative blood loss, mL	1.00 (0.99–1.00)	0.58		
Operative time, min	0.99 (0.99–1.01)	0.91		
Blood transfusion No Yes	REF 1.8 (0.60–5.93)	0.27		
Clavien-Dindo 0 1 2 3a 3b 4a 4b	REF 1.25 (0.22–6.93) 0.94 (0.21–4.14) 3.07 (0.51–18.20) - - -	0.79 0.94 0.22 0.99 0.99 0.99		
Khorana Score 1 2 3 4	REF 9.29 (1.11–77.64) 25.67 (2.79–235.65) -	0.04 0.004 0.99	REF 8.38 (0.89–78.96) 37.6 (2.43–582.35) -	0.063 0.009 -
*Abbreviations: BMI, Body Mass Index; WT, Wild Tipe; Mut, Mutated; PDS, Primary Debulking Surgery; IDS, Interval Debulking Surgery, AC, Adjuvant Chemotherapy; NACT Neoadjuvant Chemotherapy; LPS, Laparoscopy; LPT, Laparotomy; SCSS, Surgical Complexity Score System; RT, Residual Tumor—Optimal cytoreduction < 1 cm—Suboptimal cytoreduction > 1 cm; ICU, Intensive Care Unit; REF, Reference*				

## Data Availability

The data presented in this study are available on request from the corresponding author due to institutional data sharing policies and the need to ensure appropriate use within the scope of the approved research objectives.
